# Cold agglutinin disease following SARS‐CoV‐2 and *Mycoplasma pneumoniae* co‐infections

**DOI:** 10.1002/ccr3.3152

**Published:** 2020-07-20

**Authors:** Chatphatai Moonla, Phandee Watanaboonyongcharoen, Gompol Suwanpimolkul, Leilani Paitoonpong, Watsamon Jantarabenjakul, Chantiya Chanswangphuwana, Chantana Polprasert, Ponlapat Rojnuckarin, Opass Putcharoen

**Affiliations:** ^1^ Department of Medicine Faculty of Medicine Chulalongkorn University and King Chulalongkorn Memorial Hospital Thai Red Cross Society Bangkok Thailand; ^2^ Research Unit in Translational Hematology Department of Medicine King Chulalongkorn Memorial Hospital Thai Red Cross Society Bangkok Thailand; ^3^ Department of Laboratory Medicine Faculty of Medicine Chulalongkorn University and King Chulalongkorn Memorial Hospital Thai Red Cross Society Bangkok Thailand; ^4^ Thai Red Cross Emerging Infectious Diseases Clinical Center King Chulalongkorn Memorial Hospital Thai Red Cross Society Bangkok Thailand; ^5^ Department of Pediatrics Faculty of Medicine Chulalongkorn University and King Chulalongkorn Memorial Hospital Thai Red Cross Society Bangkok Thailand

**Keywords:** cold agglutinin disease, COVID‐19, hemolytic anemia, *Mycoplasma pneumoniae*, SARS‐CoV‐2

## Abstract

SARS‐CoV‐2 and other respiratory co‐infections may occur. As *Mycoplasma pneumoniae* and various viruses can cause cold agglutinin disease (CAD), the presence of CAD in COVID‐19 patients should indicate the need of investigations for those pathogens.

## Graphical abstract

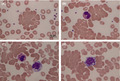

## 1 BACKGROUND

During coronavirus disease 2019 (COVID‐19) pandemic, co‐infections with other viral infections are not uncommon, but concomitant atypical bacteria are rare. Herein, we describe a young female COVID‐19 patient who developed acute cold agglutinin disease secondary to *Mycoplasma pneumoniae*. Using an azithromycin‐containing COVID‐19 therapeutic regimen, both pneumonia and anemia resolved uneventfully.

Since December 2019, COVID‐19 caused by severe acute respiratory syndrome coronavirus 2 (SARS‐CoV‐2) infection has affected millions of people globally resulting in approximately 1%‐2% case fatality.^1^ As COVID‐19 symptoms are similar to those from other respiratory pathogens, simultaneous infections with the other respiratory viruses, such as respiratory syncytial virus, enterovirus, and parainfluenza virus, occur in 20.7% (24/116) in a report from California,^2^ but appear to be rare in a report from Wuhan, China (0/99).^3^ Similarly, concomitant respiratory viral infection was not detectable in 18 COVID‐19 patients who were tested at our center in Thailand. However, the co‐infections with atypical bacteria including *M. pneumoniae* and *Chlamydia pneumoniae* are reported to be rare in the United States^2^ and Spain.^4^ Recently, Fan et al reported a COVID‐19 and *M. pneumoniae* co‐infected Singaporean patient with detectable cold agglutinins but no significant hemolysis.^5^ In this report, we described another case of similar co‐infections with a presentation of cold‐type autoimmune hemolytic anemia (AIHA).

## 2 CASE REPORT

A 24‐year‐old previously healthy Thai woman who had been studying in the UK developed fever, dyspnea, and dry cough within 24 hours after returning to Bangkok. Chest radiographs demonstrated ground‐glass opacities in the left lung at perihilar and mid‐lower regions. Reverse transcriptase‐polymerase chain reactions (RT‐PCR) could identify SARS‐CoV‐2 form her naso‐ and oropharyngeal secretions, while the other respiratory viral pathogens were undetectable by the multiplex PCR. Consequently, oral favipiravir (2 doses of 1600 mg every 12 hours followed by 600 mg twice a day for 9 days), darunavir/ritonavir (900/100 mg once a day for 10 days), hydroxychloroquine (200 mg thrice a day for 10 days), and azithromycin (single dose of 500 mg followed by 250 mg once a day for 4 days)^6^ were prescribed to treat COVID‐19 pneumonia.

**FIGURE 1:**
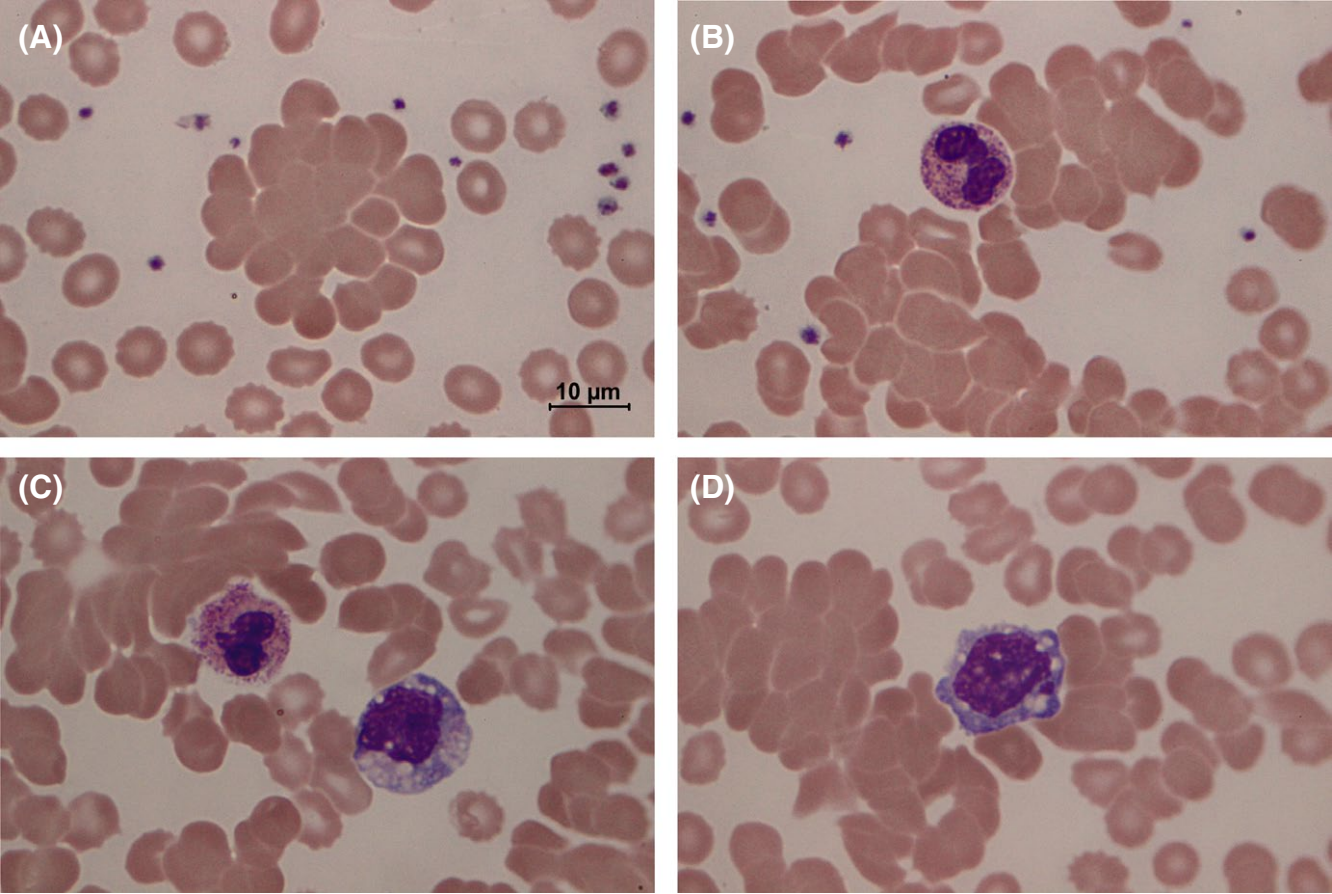
The Wright‐stained peripheral blood smear at 1000× magnification displays significant autoagglutination of erythrocytes (A‐D) as a pathognomonic feature of cold agglutinin disease. Active neutrophils and monocytes express intracytoplasmic toxic granules (B‐C) and vacuoles (C‐D) during the concurrent infections by SARS‐CoV‐2 and *Mycoplasma pneumoniae*

On the third day of the admission, there was a rapid drop of hemoglobin from baseline of 13.4 to 10.9 g/dL with markedly decreased hematocrit (26.1%) and elevated mean corpuscular hemoglobin concentration (MCHC, 41.8 g/dL). The numbers of leukocytes (5.1 × 10^3^/µL) and platelets (257 × 10^3^/µL) were normal despite mild lymphopenia (780 cells/µL) and monocytosis (1030 cells/µL). The level of serum lactate dehydrogenase (LDH) was elevated (352 U/L, normal range 125‐220 U/L). The peripheral blood smear (PBS) displayed normochromic normocytic erythrocytes with numerous red cell agglutinations, spherocytes, and microspherocytes (Figure 1). Neutrophils showed increased cytoplasmic toxic granules (Figure 1, Panel B‐C), while monocytes elicited active cytoplasmic vacuolization (Figure 1, Panel C‐D).

Her blood group was A RhD‐positive. Due to spontaneous agglutination at the room temperature, the blood sample was warmed at 37°C before testing with rabbit anti‐human globulin against immunoglobulin (Ig) G, IgA, IgM, and complement component 3 (C3: C3c and C3d) using gel cards. The direct antiglobulin test (DAT) was positive with anti‐C3d (2+) but negative with anti‐IgG, anti‐IgA, and anti‐IgM. The standard indirect antiglobulin test (IAT) was negative. The direct agglutination between I‐positive adult group O cells and patient's plasma reading reaction at the room temperature was positive, while that between I‐negative cord blood group O cells and patient's plasma was negative. Therefore, autoantibodies against I antigen (auto‐anti‐I) could be identified. On day 5 of hospitalization, cold agglutinins, and antibodies against *M. pneumoniae*, IgG and IgM, were detectable at the titers of 1:128 and 1:2560, respectively. Her hemoglobin levels were spontaneously raised from 10.9 to 12.3 g/dL in 5 days, while her respiratory condition recovered in 2 weeks with no progression to respiratory failure.

## 3 DISCUSSION

*Mycoplasma pneumoniae* is a well‐known etiology of benign and self‐remitting cold agglutinin disease (CAD) from auto‐anti‐I antibodies.^7^ Some viruses, such as Epstein‐Barr, influenza, varicella zoster, and human immunodeficiency viruses, may be linked to the transient CAD,^7^ but none of them belong to the family *Coronaviridae*. Since anemia,^1^ particularly AIHA,^8^ is uncommon in SARS‐CoV‐2 infection, cold agglutinins with or without hemolysis following COVID‐19 diagnosis may suggest a concurrent infection by *M. pneumoniae* as described in this case and the other by Fan et al.^5^

The pathogenesis of anemia in COVID‐19 patients would be associated with anemia of inflammation^9^ and microangiopathic hemolytic anemia (MAHA) due to disseminated intravascular coagulation (DIC).^10^ However, a discrepancy between hemoglobin and hematocrit reduction, and a high MCHC, which suggest red cell agglutination,^11^ should not be found in those conditions. In this case, agglutinated erythrocytes with microspherocytes of varying sizes indicating immune‐mediated hemolysis were clearly visualized on the PBS (Figure 1), although fragmented erythrocytes or schistocytes of MAHA blood picture supporting DIC were not observed. Hence, the direct examination of the PBS in every COVID‐19 case with anemia is crucial. The characteristic changes in erythrocyte morphology should guide attending physicians in proper investigations to confirm the correct diagnosis.

Our patient might gain a benefit from oral azithromycin as part of the multidrug regimen for COVID‐19 prior to the diagnosis of the co‐infection. The rationale came from the results of a prospective study showing that the addition of azithromycin to hydroxychloroquine was more effective in SARS‐CoV‐2 elimination from nasopharyngeal secretion.^6^ However, this 2‐drug combination may increase cardiovascular mortality due to their synergistic effects on QTc prolongation.^12^, ^13^, ^14^, ^15^ Supporting by the reportedly low rate of concomitant *M. pneumoniae* infection (range 0%‐0.97%),^2^, ^4^ this regimen is no longer recommended to be routinely used for COVID‐19. Nevertheless, since *M. pneumoniae* pneumonia itself requires a specific antimicrobial treatment and may increase mortality up to 1.4% in the community‐acquired setting,^16^ the administration of macrolides (azithromycin, clarithromycin, or erythromycin) or fluoroquinolones^17^ ought not to be delayed to any COVID‐19 patients who are in suspicion of concurrent infections with atypical bacteria. Moreover, due to the conflicting data regarding the frequencies and organism types of co‐infections in SARS‐CoV‐2‐infected patients,^2^, ^3^, ^4^ further microbiological investigations from various geographical locations are warranted.

In conclusion, CAD or cold agglutinin without hemolysis may be a clinical feature that suggests investigations and/or empirical antimicrobial therapy for *M. pneumoniae* in COVID‐19 patients.

## CONFLICT OF INTEREST

All authors stated that they had no interests, which might be perceived as posing a conflict or bias.

## AUTHOR CONTRIBUTIONS

CM: collected and analyzed all clinical and laboratory data, and wrote the first draft of the manuscript; PW: collected and analyzed the data of red cell antibodies; GS, LP, WJ, and OP: were involved in the diagnosis and treatment for COVID‐19 and designed the treatment protocol; CC, CP, and PR: analyzed the diagnosis for CAD and *M. pneumoniae* infection; all authors revised the manuscript.

## ETHICAL APPROVAL

All procedures performed were in accordance with the ethical standards of the institutional research committee of Faculty of Medicine, Chulalongkorn University, and with the 1964 Declaration of Helsinki.

## INFORMED CONSENT

Informed consent was obtained from the patient for the diagnostic and treatment procedures.
